# Cardiorespiratory fitness does not offset the increased risk of chronic obstructive pulmonary disease attributed to smoking: a cohort study

**DOI:** 10.1007/s10654-021-00835-4

**Published:** 2022-02-05

**Authors:** Setor K. Kunutsor, Sae Young Jae, Timo H. Mäkikallio, Jari A. Laukkanen

**Affiliations:** 1 National Institute for Health Research Bristol Biomedical Research Centre, University Hospitals Bristol and Weston NHS Foundation Trust and the University of Bristol, Bristol, UK; 2 Musculoskeletal Research Unit, Translational Health Sciences, Bristol Medical School, University of Bristol, Learning & Research Building (Level 1), Southmead Hospital, Bristol, BS10 5NB UK; 3 Department of Medicine, Central Finland Health Care District Hospital District, Finland District, Jyväskylä, Finland; 4 Department of Sport Science, University of Seoul, Seoul, Republic of Korea; 5 Department of Medicine, University of Helsinki, Helsinki, Finland; 6 Department of Medicine, South-Karelia Central Hospital, Lappeenranta, Finland; 7 Institute of Public Health and Clinical Nutrition, University of Eastern Finland, Kuopio, Finland; 8 Department of Medicine, Institute of Clinical Medicine, University of Eastern Finland, Kuopio, Finland

**Keywords:** Smoking, Cardiorespiratory fitness, Chronic obstructive pulmonary disease, Cohort study

## Abstract

Though evidence suggests that higher cardiorespiratory fitness (CRF) levels can offset the adverse effects of other risk factors, it is unknown if CRF offsets the increased risk of chronic obstructive pulmonary disease (COPD) due to smoking. We aimed to evaluate the combined effects of smoking status and CRF on incident COPD risk using a prospective cohort of 2295 middle-aged and older Finnish men. Peak oxygen uptake, assessed with a respiratory gas exchange analyzer, was used as a measure of CRF. Smoking status was self-reported. CRF was categorised as low and high based on median cutoffs, whereas smoking status was classified into smokers and non-smokers. Multivariable-adjusted hazard ratios with confidence intervals (CIs) were calculated. During 26 years median follow-up, 119 COPD cases were recorded. Smoking increased COPD risk 10.59 (95% CI 6.64–16.88), and high CRF levels decreased COPD risk 0.43 (95% CI 0.25–0.73). Compared with non-smoker-low CRF, smoker-low CRF was associated with an increased COPD risk in multivariable analysis 9.79 (95% CI 5.61–17.08), with attenuated but persisting evidence of an association for smoker-high CRF and COPD risk 6.10 (95% CI 3.22–11.57). An additive interaction was found between smoking status and CRF (RERI = 6.99). Except for CRF and COPD risk, all associations persisted on accounting for mortality as a competing risk event. Despite a wealth of evidence on the ability of high CRF to offset the adverse effects of other risk factors, it appears high CRF levels have only modest attenuating effects on the very strong association between smoking and COPD risk.

**Supplementary Information:**

The online version contains supplementary material available at 10.1007/s10654-021-00835-4.

## Introduction

Chronic obstructive pulmonary disease (COPD) is a chronic inflammatory disease of the lungs that results in progressive and irreversible airflow obstruction [1]. It is the third leading cause of death globally; there were 3.23 million COPD-related deaths in 2019 [2]. Apart from being one of the leading causes of death, COPD is associated with substantial healthcare costs and recurrent hospitalizations and also a major cause of disability-adjusted life years [1, 3]. COPD represents three percent of the healthcare spending in Europe [4]. Though active smoking is the major risk factor for COPD, not all smokers develop COPD—it has been reported that 20–25% of smokers develop COPD [1]. The prevalence of COPD in non-smokers has been estimated to be 4%, suggesting the existence of other risk factors [3]. Other important risk factors include occupational exposure (e.g., dust, fumes, chemicals), indoor air pollution, and infections [2, 3]. Though COPD is incurable, early diagnosis and treatment can slow the progression of symptoms. In addition, COPD is potentially preventable through modulation or reduction in exposure to underlying risk factors.

Cardiorespiratory fitness (CRF), often expressed as maximal oxygen consumption (VO_2_ max) in healthy individuals or peak VO_2_ in those with limitations to exercise, is a modifiable risk factor that can be improved through exercise training and increased physical activity [5], which is associated with reduced risk of COPD [6]. CRF is an independent predictor for all-cause and disease-specific morbidity and mortality [5]. High CRF levels have also been demonstrated to be associated a lower risk of respiratory diseases including incident COPD and death from COPD [7, 8]. CRF, which is dependent on both cardiovascular and pulmonary function, has recently been proposed as a vital sign and reported to be stronger than many traditional risk factors for COPD, such as type 2 diabetes mellitus and smoking [5]. There is growing consistent evidence that higher levels of CRF can attenuate or offset the adverse effects of other risk factors [9–11]. Furthermore, studies have demonstrated the protective effect of higher CRF against smoking-related cancer incidence and mortality [12, 13]. Given the overall evidence, we hypothesized that CRF could offset the increased risk of COPD due to smoking. In this context, we aimed to evaluate the combined effects of smoking status and CRF on the risk of incident COPD using a population-based prospective cohort of 2295 middle-aged and older Finnish men. We also evaluated the separate associations of smoking status and CRF with the risk of COPD to confirm previous evidence of the associations.

## Methods

The study population was part of the Kuopio Ischemic Heart Disease (KIHD) population-based prospective cohort study, comprising a representative sample of middle-aged and older men aged 42–61 years recruited from Kuopio, eastern Finland. They had baseline examinations performed from March 1984 through December 1989. The study was approved by the Research Ethics Committee of the University of Eastern Finland, and each participant gave written informed consent. Participants completed self-administered health and lifestyle questionnaire for the assessment of smoking and other factors. Smoking was categorised as smokers and non-smokers. A participant was defined as a smoker if he had ever smoked regularly and had smoked cigarettes, cigars, or a pipe within the past 30 days. Peak oxygen uptake (VO_2peak_) was used as a measure of CRF, which was directly assessed using a computerized metabolic measurement system (Medical Graphics, MCG, St. Paul, Minnesota) during progressive exercise testing to volitional fatigue on an electrically braked cycle ergometer [14]. The standardized testing protocol included a 3-min warm-up at 50 watts (W); 1 W = 6.12 kg/min), followed by 20 W/min increases in workload with direct analyses of expired respiratory gases. We included all incident cases of COPD that occurred from study enrollment through 2014. No losses to follow-up were recorded in the KIHD study. Participants (using Finnish personal identification codes) are under continuous annual surveillance for the development of new outcome events. Incident COPD cases were collected by linkage to the National Hospital Discharge Register. Qualified physicians made the diagnoses of COPD which was based on clinical history, symptoms and spirometry findings (based on forced expiratory volume in 1 s (FEV1) and forced vital capacity (FVC)). The FEV1/FVC ratio < 0.70 of the participants’ best reading was used as the threshold for expiratory airway obstruction, i.e., COPD.

Multivariable-adjusted hazard ratios (HRs) with 95% confidence intervals (CIs) for incident COPD were estimated using Cox proportional hazard models. Selection of confounders was based on their previously established role as risk factors for COPD, evidence from previous research, or their potential as confounders based on known associations with COPD and observed associations with the exposures using the available data [15]. CRF was modelled as both categorical (tertiles) and continuous (per standard deviation (SD) increase) variables. Evaluation of the joint association of smoking status and CRF with COPD risk was based on the following four combinations of smoking status categories and median cutoffs for CRF: non-smoker-low CRF; non-smoker-high CRF; smoker-low CRF; and smoker-high CRF. Formal tests of interaction were used to assess if the two exposures are independent on the risk of COPD. Interactions between smoking status and CRF were examined on both the additive and multiplicative scales in relation to COPD risk. Additive interactions were assessed using the “relative excess risk due to interaction” (RERI), computed for binary variables as RERI_HR_ = HR_11_ − HR_10_ − HR_01_ + 1 [16]. Multiplicative interactions were assessed using the ratio of HRs = HR_11_/(HR_10_xHR_01_) [16]. A positive additive interaction is indicated if RERI > 0 and a positive multiplicative interaction is indicated if the ratio of HRs > 1. All statistical analyses were conducted using Stata version MP 17 (Stata Corp, College Station, Texas).

## Results

**Fig. 1:**
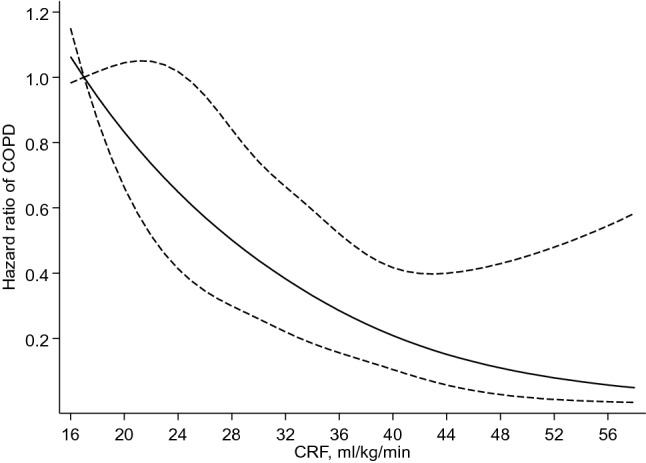
Restricted cubic spline of the hazard ratios of incident chronic obstructive pulmonary disease with cardiorespiratory fitness**.** Reference value for cardiorespiratory fitness is 17 ml/kg/min; dashed lines represent the 95% CIs for the spline model (solid line). Models were adjusted for age, body mass index, history of type 2 diabetes, prevalent coronary heart disease, history of asthma, history of chronic bronchitis, history of tuberculosis, alcohol consumption, energy intake, leisure-time physical activity, and socioeconomic status**.** *COPD* chronic obstructive pulmonary disease

The overall mean (SD) age and CRF of study participants at baseline was 53 (5) years and 30.3 (8.0) ml/kg/min, respectively (Table 1). Age-standardized values of CRF based on methods previously suggested [17] are provided in Appendix 1. Smokers comprised 31.5% (723) of the study participants. During a median (interquartile range) follow-up of 26.0 (18.3–28.0) years, 119 incident cases of COPD occurred. Compared to non-smokers, smokers had an increased risk of COPD following adjustment for age, body mass index, history of type 2 diabetes, histories of coronary heart disease, asthma, chronic bronchitis and tuberculosis, alcohol consumption, energy intake, leisure-time physical activity, and socioeconomic status 11.39 (95% CI 7.16–18.11), which was minimally attenuated to 10.59 (95% CI 6.64–16.88) following further adjustment for CRF. A multivariable restricted cubic spline curve showed that the risk of COPD decreased continuously with increasing CRF across the range 25–65 ml/kg/min (*P*-value for nonlinearity = 0.95) (Fig. 1). The HR for COPD per 1 SD increase in CRF in analysis adjusted for the covariates above plus smoking status was 0.66 (95% CI 0.52–0.84) (Table 2). When the top tertile of CRF was compared to the bottom tertile, the corresponding HR for COPD was 0.43 (95% CI 0.25–0.73) (Table 2).

**Table 1:** Baseline characteristics of study participants

Characteristics	Mean (SD) or median (IQR) or n (%)
Cardiorespiratory fitness, ml/kg/min	30.3 (8.0)
Age	
*Questionnaire/prevalent conditions*	
Age, year	53 (5)
Alcohol consumption, g/week	31.5 (6.4–92.3)
Total energy intake, kJ/day	9919 (2589)
Leisure-time physical activity, kJ/day	1208 (631–1991)
History of type 2 diabetes	80 (3.5)
Current smoking	723 (31.5)
History of CHD	541 (23.6)
History of asthma	77 (3.4)
History of chronic bronchitis	163 (7.1)
History of tuberculosis	87 (3.8)
*Physical measurements*	
BMI, kg/m^2^	26.9 (3.5)
SBP, mmHg	134 (17)
DBP, mmHg	89 (10)
Socio-economic status	8.43 (4.25)
*Blood biomarkers*	
Total cholesterol, mmol/l	5.91 (1.07)
HDL-C, mmol/l	1.29 (0.30)
Fasting plasma glucose, mmol/l	5.34 (1.32)

*BMI* body mass index, *CHD* coronary heart disease, *DBP* diastolic blood pressure, *HDL-C* high-density lipoprotein cholesterol, *IQR* interquartile range, *SD* standard deviation, *SBP* systolic blood pressure

**Table 2:** Separate and combined associations of smoking status and cardiorespiratory fitness with the risk of chronic obstructive pulmonary disease

Exposure categories	Events/total	Model 1		Model 2		Model 3		Model 4	
		HR (95% CI)	*P-*value	HR (95% CI)	*P-*value	HR (95% CI)	*P-*value	HR (95% CI)	*P-*value
*Smoking status*									
Non-smoker	25/1572	Ref.		Ref.		Ref.		Ref.	
Smoker	94/723	12.57 (8.07–19.59)	< 0.001	11.39 (7.16–18.11)	< 0.001	10.59 (6.64–16.88)	< 0.001	7.72 (3.40–17.54)	< 0.001
*CRF (ml/kg/min)*									
Per 1 SD increase in CRF	119/2295	0.60 (0.48–0.74)	< 0.001	0.59 (0.46–0.74)	< 0.001	0.66 (0.52–0.84)	0.001	0.74 (0.50–1.10)	0.13
Tertile 1 (6.4–26.8)	62/765	Ref.		Ref.		Ref.		Ref.	
Tertile 2 (26.9–33.2)	32/765	0.46 (0.30–0.71)	< 0.001	0.49 (0.31–0.76)	0.002	0.49 (0.31–0.77)	0.002	0.72 (0.29–1.79)	0.48
Tertile 3 (33.3–65.0)	25/765	0.36 (0.22–0.58)	< 0.001	0.34 (0.20–0.58)	< 0.001	0.43 (0.25–0.73)	0.002	0.61 (0.20–1.81)	0.37
*Smoking status and CRF (ml/kg/min) combination*									
Non-smoker-low CRF	17/733	Ref.		Ref.		NA	NA	Ref.	
Non-smoker-high CRF	8/839	0.43 (0.18–1.00)	0.05	0.45 (0.19–1.07)	0.07	NA	NA	0.28 (0.06–1.27)	0.10
Smoker-low CRF	60/415	10.11 (5.88–17.37)	< 0.001	9.79 (5.61–17.08)	< 0.001	NA	NA	4.89 (1.82–13.15)	0.002
Smoker-high CRF	34/308	7.13 (3.92–12.97)	< 0.001	6.10 (3.22–11.57)	< 0.001	NA	NA	4.44 (1.42–13.89)	0.01

*CI* confidence interval, *CRF* cardiorespiratory fitness, *HR* hazard ratio, *NA* not applicable, *ref* reference, *SD* standard deviation

Model 1: adjusted for age

Model 2: model 1 plus body mass index, history of type 2 diabetes, prevalent coronary heart disease, history of asthma, history of chronic bronchitis, history of tuberculosis, alcohol consumption, energy intake, leisure-time physical activity, and socioeconomic status

Model 3: model 2 plus CRF for smoking status and smoking status for CRF

Model 4: model 3 plus all-cause mortality as a competing risk event

**Fig. 2:**
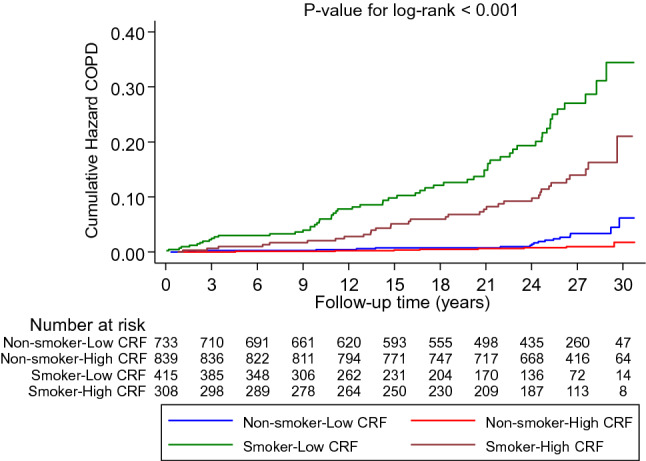
Crude cumulative Kaplan–Meier curves for COPD during follow-up according to combined categories of smoking status and CRF. *COPD* chronic obstructive pulmonary disease, *CRF* cardiorespiratory fitness

Crude cumulative hazard curves showed the risk for COPD was highest for smoker-low CRF group compared with other groups (*P*-value for log-rank test < 0.001; Fig. 2). Compared with non-smoker-low CRF, smoker-low CRF was associated with an increased risk of COPD in multivariable analysis 9.79 (95% CI 5.61–17.08), with attenuated but persisting evidence of an association for smoker-high CRF and COPD risk 6.10 (95% CI 3.22–11.57). Results of interaction analysis showed the RERI was 6.99 and the ratio of HRs was 0.72, indicating the presence of an additive interaction but absence of a multiplicative interaction.

Given the high mortality rate in the KIHD cohort, we included a fourth model in the association analysis to estimate the baseline cumulative subhazard of COPD considering all-cause mortality as a competing outcome to COPD. A total of 1112 deaths occurred during follow-up. In analyses including all-cause mortality as a competing risk event, there was still significant evidence of associations of smoking status and smoking status-CRF combinations (smoker-low CRF and smoker-high CRF) with COPD risk, but the association was attenuated to null for CRF and COPD risk (Table 2).

To minimize the effects of potential reverse causation, we re-analysed the data on exclusion of the first five years of follow-up and the findings were similar to the main results (Appendix 2).

## Comment

Our findings showed that smokers were about 11 times as likely to develop COPD than non-smokers, findings which confirm the well-established fact that smoking is the major risk factor for COPD. We have also confirmed the independent associations of elevated levels of CRF with a decreased risk of COPD, which was consistent with a linear dose–response relationship. New findings based on the joint associations of smoking status and CRF with the risk of COPD showed that the risk of COPD was substantially increased in men who were smokers and had low CRF levels; however, the substantial risk of COPD due to smoking was slightly attenuated but persisted in the presence of high CRF levels. Formal analysis showed significant evidence of interactive effects of smoking status and CRF (on the additive scale) on the long-term risk of COPD. Given the high mortality rate in our study cohort which might have hindered our event of interest, the association between CRF and COPD was less robust when all-cause mortality was adjusted for as a competing risk event. This was not a surprising finding as CRF was independently associated with all-cause mortality in the cohort (findings not shown). All findings remained similar on exclusion of the first five years of follow-up.

Physical fitness, a strong predictor of future health status [18], has CRF and muscular fitness as its main components [19]. High levels of CRF have well-established health benefits and the ability to modify or offset the adverse effects of other risk factors. The current findings show that high CRF levels do have interactive and attenuating effects on the association between smoking and COPD risk, but these effects are only modest. This could be due to the fact that the relationship between smoking and COPD is very strong and causal [20]. A substantial proportion of COPD cases are caused by other factors such as occupational exposure, environmental tobacco smoke, indoor air pollution, and genetic factors, but their contribution is much less than active smoking. Though the chronic inflammatory process in COPD persists after smoking cessation, the most clinically and cost-effective treatment for COPD is still smoking cessation [20].

Our evaluation of the separate effects of CRF levels on COPD risk showed that high CRF levels could confer protective effects irrespective of smoking status. Taking the whole evidence together, it can be postulated that CRF has the potential to reduce the risk of COPD, but its effect is modest in the presence of active smoking. Like other adverse chronic outcomes, the beneficial effects of CRF on COPD may be exerted via the effects of regular physical activity. In addition to the anti-inflammatory effects of habitual physical activity [21], it has direct effects which include increasing the amount of ventilation in pulmonary airways and reducing lung function decline, hence preventing or delaying the onset of COPD [22].

The strengths of this study include the evaluation of the combined effects of smoking status and fitness levels, utilization of a large sample, the prospective cohort design with long follow-up, and the direct measurement of VO_2peak_ using expired gas analysis, which provides a gold standard measure of CRF. We had access to a comprehensive panel of relevant confounders which allowed adequate adjustment and we were able account for the effect of reverse causation. Our limitations were mainly inherent and included the lack of granular data on smoking status which was only categorized into smokers and non-smokers, inability to generalize the findings to women and other ethnicities and the potential for biases such as residual confounding and regression dilution. Our reproducibility substudies of CRF measurements within the KIHD study show high within-person variability in CRF levels measured many years apart [23] which suggests the risk estimates observed in the current analysis are underestimated.

In conclusion, both smoking status and CRF are each independently associated with COPD risk in middle-aged and older Finnish men. Contrary to previous evidence on the ability of high CRF levels to offset the adverse effects of other chronic risk factors, high CRF levels have only modest attenuating effects on the heightened risk of COPD due to active smoking. The association between smoking and COPD risk is very strong and may only be modestly attenuated by lifestyle changes that increase CRF, such as increased exercise training and physical activity.

## Supplementary Information

Below is the link to the electronic supplementary material.

Supplementary file1 (DOCX 23 kb)

## Data Availability

The data used for this study are available from the corresponding author upon reasonable request.
