# Supplementary material for: Cardiorespiratory fitness does not offset the increased risk of chronic obstructive pulmonary disease attributed to smoking: a cohort study
Source: Eur J Epidemiol. 2022 Feb 5;37(4):423–8. doi: 10.1007/s10654-021-00835-4 (PMC9187537; doi:10.1007/s10654-021-00835-4)
Supplement: Supplementary file 1 — Supplementary file1 (DOCX 23 kb) [file 10654_2021_835_MOESM1_ESM.docx]

**Supplementary Material**

| **Appendix 1** | Values and categories of age-specific cardiorespiratory fitness |
| --- | --- |
| **Appendix 2** | Separate and combined associations of smoking status and cardiorespiratory fitness with the risk of chronic obstructive pulmonary disease, on exclusion of the first 5 years of follow-up |

**Appendix 1.** Values and categories of age-specific cardiorespiratory fitness

| **Age category, yrs** | **n** | **Mean (SD)** | **Tertile 1** | **Tertile 2** | **Tertile 3** | **Below median** | **Above median** |
| --- | --- | --- | --- | --- | --- | --- | --- |
| 42-47 | 296 | 35.5 (7.5) | <32.0 | 32.0-38.1 | 38.2-58.3 | < 35.0 | ≥35.0 |
| 48-53 | 305 | 33.2 (7.5) | < 29.7 | 29.7-35.7 | 35.8-57.3 | < 32.2 | ≥32.2 |
| 54-59 | 1398 | 29.6 (7.6) | < 26.4 | 26.4-32.5 | 32.6-65.4 | < 29.4 | ≥29.4 |
| ≥60 | 296 | 25.1 (6.8) | < 22.6 | 22.6-27.8 | 27.9-47.8 | < 25.2 | ≥25.2 |

SD, standard deviation

**Appendix 2.** Separate and combined associations of smoking status and cardiorespiratory fitness with the risk of chronic obstructive pulmonary disease, on exclusion of the first 5 years of follow-up

| **Exposure categories** | **Events/**  **Total** | **Model 1** | | **Model 2** | | **Model 3** | | **Model 4** | |
| --- | --- | --- | --- | --- | --- | --- | --- | --- | --- |
|  |  | HR (95% CI) | *P-*value | HR (95% CI) | *P-*value | HR (95% CI) | *P-*value | HR (95% CI) | *P-*value |
| **Smoking status** | | | | | | | | | |
| Non-smoker | 22 / 1521 | ref |  | ref |  | ref |  | ref |  |
| Smoker | 79 / 652 | 12.59 (7.83-20.24) | < .001 | 11.31 (6.89-18.56) | <.001 | 10.71 (6.51-17.63) | <.001 | 7.60 (3.33-17.36) | <.001 |
| **CRF (ml/kg/min)** | | | | | | | | | |
| Per 1 SD increase in CRF | 101 / 2173 | 0.64 (0.51-0.80) | <.001 | 0.63 (0.49-0.81) | <.001 | 0.71 (0.54-0.92) | .01 | 0.71 (0.47-1.08) | .11 |
| Tertile 1 (6.4-26.8) | 49 / 725 | ref |  | ref |  | ref |  | ref |  |
| Tertile 2 (26.9-33.2) | 31 / 724 | 0.57 (0.36-0.90) | .015 | 0.60 (0.38-0.97) | .037 | 0.57 (0.35-0.91) | .018 | 0.81 (0.32-2.04) | .66 |
| Tertile 3 (33.3-65.0) | 21 / 724 | 0.38 (0.22-0.65) | <.001 | 0.36 (0.20-0.65) | .001 | 0.44 (0.24-0.79) | .006 | 0.61 (0.20-1.85) | .38 |
| **Smoking status and CRF (ml/kg/min) combination** | | | | | | | | | |
| Non-smoker-Low CRF | 15 / 717 | ref |  | ref |  | NA | NA | ref |  |
| Non-smoker-High CRF | 7 / 804 | 0.43 (0.17-1.06) | .07 | 0.45 (0.18-1.13) | .09 | NA | NA | 0.29 (0.06-1.38) | .12 |
| Smoker-Low CRF | 49 / 370 | 10.01 (5.60-17.89) | <.001 | 9.53 (5.25-17.29) | <.001 | NA | NA | 4.70 (1.70-13.00) | .003 |
| Smoker-High CRF | 30 / 282 | 7.34 (3.88-13.89) | <.001 | 6.32 (3.19-12.54) | <.001 | NA | NA | 4.67 (1.43-15.22) | .01 |

CI, confidence interval; CRF, cardiorespiratory fitness; HR, hazard ratio; NA, not applicable; ref, reference; SD, standard deviation

Model 1: Adjusted for age

Model 2: Model 1 plus body mass index, history of type 2 diabetes, prevalent coronary heart disease, history of asthma, history of chronic bronchitis, history of tuberculosis, alcohol consumption, energy intake, and leisure-time physical activity, socioeconomic status

Model 3: Model 2 plus CRF for smoking status and smoking status for CRF

Model 4: Model 3 plus all-cause mortality as a competing risk event
